# Human SUMOylation Pathway Is Critical for Influenza B Virus

**DOI:** 10.3390/v14020314

**Published:** 2022-02-03

**Authors:** Runrui Dang, Victor G. J. Rodgers, Adolfo García-Sastre, Jiayu Liao

**Affiliations:** 1 Department of Bioengineering, Institute for Integrative Genome Biology, School of Medicine, College of Engineering, Biomedical Science, University of California at Riverside, 900 University Avenue, Riverside, CA 92521, USA; rdang018@ucr.edu (R.D.); vrodgers@engr.ucr.edu (V.G.J.R.); 2 Department of Microbiology, Global Health and Emerging Pathogens Institute, 1468 Madison Avenue, New York, NY 10029, USA; Adolfo.Garcia-Sastre@mssm.edu; 3 Department of Medicine, Division of Infectious Diseases, 1468 Madison Avenue, New York, NY 10029, USA; 4 Department of Pathology, Molecular and Cell-Based Medicine, Icahn School of Medicine at Mount Sinai, 1468 Madison Avenue, New York, NY 10029, USA

**Keywords:** influenza B virus or IBV, critical host factor, SUMOylation, therapeutics

## Abstract

The identification and elucidation of host pathways for viral infection are critical for understanding the viral infection processes and novel therapeutics development. Here, for the first time, we discover that the human SUMOylation pathway is essential for the IBV viral life cycle. First, IBV viruses were completely inhibited by a novel SUMOylation specific inhibitor, STE025, discovered from our FRET-based high-throughput screening, and the inhibition was very potent, with IC_50_~ 0.1 µM in an IBV-induced cell death rescue assay; Second, we determined that the IBV M1 protein was SUMOylated, which was mediated by the SUMOylation E2 conjugation enzyme and the E3 ligase enzyme at very high affinities, of 0.20 µM and 0.22 µM, respectively; Third, the mutation of the IBV M1 SUMOylation site, K21R, completely abolished the viral particle generation, strongly suggesting the requirement of SUMOylation for the IBV life cycle. These results suggest that the blockage of the host human SUMOylation pathway is very effective for IBV inhibition. We therefore propose that the host SUMOylation pathway is a critical host factor for the IBV virus life cycle. The identification and inhibition of critical host factor(s) provide a novel strategy for future anti-viral therapeutics development, such as IBV and other viruses.

## 1. Introduction

Influenza A and B viruses (IAV and IBV, respectively, or flu) have caused several outbreaks in history, including the recent 2009 H1N1 influenza pandemic, and causes a significant number of deaths every year despite significant advancements in our understanding of the viruses and the availability of vaccines and antiviral agents. In the season of 2017–2018, it is estimated that flu caused approximately 51,000 deaths and 710,000 hospitalizations, making it the most life-threatening infectious disease [1]. The estimated average yearly economic burden of influenza is $11.2 billion, including $3.2 billion in direct costs and $8.0 billion in indirect costs annually [2]. The origin of IBV was first discovered in 1940, and later circulating strains diverged into two lineages, were named in 1983 as the Yamagata and Victoria lineages [3]. Both IBV lineages have been co-circulating each influenza season, contributing significantly to the flu disease burden over the years [4]. The scientific and healthcare communities have been underestimating the roles of IBV in terms of disease burden, as IBV was believed to cause milder symptoms compared to some IAV strains. However, several studies have suggested the increased potency of influenza B virus in causing severe disease and mortality in some populations and contributing significantly to the annual disease every year, accounting for 37% of the economic costs caused by flu every year [4]. For example, it has shown that IBV can cause severe disease in children [5,6]. A recent study found significantly higher mortality rates due to IBV compared to IAV in children younger than 16 years of age from 2004 to 2013 [7]. IBV also causes higher hospitalizations in HIV patients than IAV [8]. In addition, population studies show that IBV infections have significantly higher mortality rates than influenza A infections in some flu seasons. For example, during the flu season of 2010–2011, only 26% of circulating strains of flu were influenza B viruses, but they was responsible for 38% of deaths in the pediatric population [9]. These data strongly refute the opinions that IBV causes milder symptoms than IAV.

The efforts to reduce the transmission and pathology of IBV include the introduction of a quadrivalent vaccine that includes both lineages of IBV [10]. These vaccines significantly decrease the rates of flu infection; but only a disappointingly low effectiveness was achieved in susceptible populations such as children within the age group of 9–17 years of age (43%) or elderly people with >65 years of age (34%) in the flu season of 2019–2020 and other years, indicating limitations in our current vaccine approach and need for additional therapeutic reagents [11].

There are three FDA-approved neuraminidase inhibitors (NAI) recommended for flu by the CDC: oseltamivir, zanamivir, and peramivir. Most recently, circulating influenza viruses have been susceptible to NAI antiviral medications. However, recent virus isolates from patients show significant drug resistance including IBV [12,13,14,15,16,17]. A drug resistance mutation Gly407Ser was discovered in the neuraminidase IBV Yamagata lineage [18]. The mutations in neuraminidase, such as Asp198Asn, Ile222Thr, and Ser250Gly, led to persistent viral shedding in children infected with IBV [19]. Antiviral drugs also often lead to gastrointestinal adverse effects, which makes antiviral therapies a not so easy decision in a clinical setting [20]. Recently, Baloxavir, an influenza cap-dependent endonuclease inhibitor, was approved by the FDA for treating both IAV- and IBV-infected patients [21]. However, drug resistance mutations are also expected as Baloxavir is an inhibitor of a viral protein, and the low fidelity of viral RNA polymerase is prone to error generation. Issues of antiviral resistance again emphasize the need for antiviral therapies with novel mechanisms.

As a pathogen to humans, viruses must engage host factors for their infection and replication, and therapeutics targeting host–virus interactions could present a potentially effective and broad-spectrum treatment modality for viral infections, such as IAV/IBV. Among the interactions of viruses and humans, a post-translation modification (PTM), the SUMOylation, is unique in several aspects. Several genome-wide siRNA or CRISPR screenings have identified that SUMOylation enzyme inhibition or gene knockout decreases the replication of the influenza virus [22,23,24]. In some of these screenings, a luciferase-labeled IAV virus was used for infections of cells, and luciferase activities were monitored as a surrogate of viral production. The SUMOylation genes are on the top lists from these screenings, but detailed mechanisms underlining their role on virus replication have not been fully explored. Importantly, SUMO E3 ligase gene PIAS1 knockout mice are resistant to lethal-dose infections of viruses and bacteria, such as Vesicular Stomatitis Virus (VSV) and *L. monocytogenes*, indicating an important role of SUMOylation in promoting these pathogens directly or indirectly [25]. Furthermore, many viral proteins are directly SUMOylated, such as the NS1, M1, NP, PA, and PB proteins of influenza viruses, and mutations of their SUMOylation sites have a significant impact on virus growth [26,27,28,29,30,31,32]. We recently identified critical or essential SUMOylation sites on IAV NS1 and M1 proteins, respectively, and these mutations either significantly reduce viral replication or completely abolish the virus growth [33] (Way, G., Submitted).

The influenza M1 gene plays multiple roles in the influenza A virus’ life cycle, from infection to new virion release, and such multifaceted functional roles impose significant constraints on the space of M1 to evolve. From large-scale sequencings from different hosts and geographic locations, two studies show that M1 is one of the slowest-evolving genes in the influenza genome [34,35]. In human seasonal influenza viruses, it has been shown that the evolution of M1 is 5- to 10-fold slower than that of HA genes, and even slower than that of the M2 gene [34,36]. The high degree of conservation of the M1 gene may also be due to its internal protein and is targeted by cellular immunity, such as CD4 and CD8, with not as much selection pressure as when targeted by antibodies [37,38].

Although many studies indicate various similarities between IAV and IBV, many questions have yet to be answered for influenza B. Why does influenza B show higher infectivity in children compared to adults? Do IAV and IBV share similar host pathways? In this study, we first discovered that IBV generation could be completely inhibited by our novel SUMOylation inhibitor discovered from our FRET-based high-throughput screening. We then found that the M1 protein of IBV is SUMOylated, and that the M1 is recognized by both SUMO E2 Ubc9 and E3 PIAS1 at very high affinities as determined with a quantitative FRET (qFRET) method. Furthermore, we found that the K21R mutation of the M1 protein completely abolishes viral replication, indicating the essential role of SUMOylation for M1 function in the virus cycle. We would like to emphasize that a further understanding of IBV’s dependence on SUMOylation requires further investigation. These efforts suggest that SUMOylation inhibition might be employed for the treatment of IAV and IBV infections.

## 2. Materials and Methods

### 2.1. Molecular Cloning of DNA Constructs

The pET28b (+) constructs for CyPet-SUMO1, UBA2, AOS1, and UBC9 were cloned as outlined in our previous study [39]. The Ypet-M1 was constructed by amplifying the open reading frame (ORF) of YPet with primers and a Linker sequence and ligated into pET28b (+) vector (Millipore Corporation, Billerica, MA), and primers designed to introduce site-specific mutations and amplify the full plasmid [40]. The lysine-to- arginine M1 mutants were sequenced to verify that the correct mutations were introduced. All plasmid DNA constructs were amplified in TOP10 DH5a *E. coli* cells. The plasmids used for the generation of recombinant influenza virus were first described by Fodor et al. [41].

### 2.2. Cell Lines

Human embryonic kidney 293 cells (HEK 293) and Madin–Darby canine kidney cells (MDCK) were cultivated in Dulbecco’s modified Eagles medium (DMEM, GIBCO, Carlsbad, CA) supplemented with 10% fetal bovine serum (GIBCO) and 1% pen strep glutamine 100X (GIBCO) in the incubator with 5% CO_2_ at 37 °C.

### 2.3. Protein Expression and Purification

BL21 DE3 *E. coli* cells were transformed with the pET28b (+) constructs encoding CyPet-SUMO1, AOS1, UBA2, UBC9, and YPet-Linker3-M1. The transformed *E. coli* were plated onto LB plates containing 50 mg/mL kanamycin. A single colony was inoculated into 5 mL liquid LB with 50 mg/mL kanamycin starter culture. Each starter culture was transferred into 1 L 2YT medium with 50 mg/mL kanamycin and grown at 37 °C and 180 RPM for 3 h. The expression of recombinant proteins were induced with 0.35 mM IPTG at 20 °C and 180 RPM overnight. The bacterial cells were harvested the next day at 4 °C and 8000 for 5 min. The bacterial cell pellet was resuspended in 30 mL of a binding buffer (20 mM Tris-HCl pH 7.4, 500 mM NaCl, and 5 mM imidazole). The cell suspension was lysed with an ultrasonic liquid processor (Misonix, Farmingdale, NY, USA). The supernatant was collected after centrifugation at 4 °C and 35,000× *g* for 30 min. All expression proteins with 6x His-tag were purified by Ni2+-NTA agarose beads (QIAGEN, Valencia, CA, USA). The column was washed sequentially with two column volumes of Wash Buffer 1 (20 mM Tris-HCl pH 7.4 and 300 mM NaCl), two column volumes of Wash Buffer 2 (20 mM Tris-HCl pH 7.4, 0.5% TritonX-100, and 1.5 M NaCl), and one column volume of Wash Buffer 3 (20 mM Tris-HCl pH 7.4, 500 mM NaCl, and 10 mM imidazole), and eluted with an elution buffer (20 mM Tris-HCl pH 7.4, 20 mM NaCl, and 400 mM imidazole). Recombinant proteins were dialyzed overnight at 4 °C in a dialysis buffer (20 mM Tris- HCl pH 7.4, 50 mM NaCl, and 1 mM DTT). Protein purity was determined by SDS-PAGE after Coomassie G-250 staining (Bio-Rad, Hayward, CA, USA), and protein concentrations were determined by a Bradford assay with known amounts of bovine serum albumin (Thermo-Fisher Scientific Inc., Rockford, IL, USA) as standards. The concentrations of fluorescent-fusion proteins (CyPet-SUMO1 and Ypet-Linker3-M1) were determined by fluorescence intensities measured on a FlexStationII^384^ (Molecular Devices, Sunnyvale, CA, USA).

### 2.4. qFRET Determination of the Dissociation Constant (K_D_)

The dissociation constant K_D_ was determined by setting up a series of reactions with the receptor concentration at 0.20 µM ([R]Total, CyPet binding protein SUMO1) and a varying concentration of ligand ([L]Total, YPet binding protein M1) from 0 to 4 µM. CyPet and YPet binding proteins were mixed and diluted with PBS in a total volume of 60 µL, and this step was repeat three times. The sample was heated up to 37 ℃ and incubated at 15~20 min. Then, all sample mixtures were transferred to a Greiner 384-well plate (Sigma-Aldrich). The fluorescence emissions were measured using FlexstationII384. The emission intensities were measured at three wavelengths: 475 and 530 nm after excitation at 414 nm, and 530 nm after excitation at 475 nm [42]. Then the K_D_ was calculated by our previously defined equation, which is shown in Equation (1).

(1) $$E_{mFRET}=E_{mFRET}*\frac{{\left[L\right]}_{Total}-{\left[R\right]}_{Total}-K_{D}+\sqrt{{\left({\left[R\right]}_{Total}+K_{D}-{\left[L\right]}_{Total}\right)}^{2}+4*K_{D}*{\left[L\right]}_{Total}}}{{\left[R\right]}_{Total}+K_{D}-{\left[L\right]}_{Total}+\sqrt{{\left({\left[R\right]}_{Total}-{\left[L\right]}_{Total}+K_{D}\right)}^{2}+4*K_{D}*{\left[L\right]}_{Total}}}$$

### 2.5. qFRET In Vitro SUMOylation Assay

All components of the SUMOylation assay (0.5 mM CyPet-SUMO1, 0.1 mM E1, 0.2 mM E2, 0.25 mM E3 PIAS1, and 2 mM YPet-Linker3-M1) were combined in the SUMOylation buffer containing 50 mM Tris-HCl pH 7.4, 1 mM DTT, and 4 mM MgCl_2_ in a total volume of 60 mL. The sample mixtures were an added 1 mM ATP were incubated in an Eppendorf tube at 37 °C, and all sample mixtures were transferred to the Greiner 384-well plate (Sigma-Aldrich). The fluorescence emissions were measured using FlexstationII384 (Molecular Devices, Sunnyvale, CA, USA). Emission intensities were measured at three wavelengths: 475 and 530 nm after excitation at 414 nm, and 530 nm after excitation at 475 nm [42].

### 2.6. Em_FRET_ Analysis

As described in the DNA constructs section, we fused the CyPet and YPet to the amino termini of the SUMO1 and M1, respectively. The peak wavelengths of excitation and emission were 414 nm/475 nm for CyPet and 475 nm/530 nm for YPet. When the FRET pair (CyPet and YPet) were in close (between 1–10 nm), the excitation of the donor at 414 nm resulted in an energy transfer from the donor to the acceptor, resulting in the quenching of the donor and the excitation of the acceptor. Upon the SUMOylation of YPet-M1 with a CyPet-SUMO1, FRET can occur, resulting in a 530-nm emission given a 414-nm excitation. However, anything that prevents SUMOylation (the absence of ATP or addition of STE025) would result in no increase of the emission at 530 nm.

The real FRET emission (Em_FRET_) was used to monitor the formation of the SUMO1-M1 complex. We defined EmFRET as shown in Equation (2) [42]. To obtain the real FRET emission, which is correlated with the amount of bound CyPet-SUMO1 and YPet- M1, the direct emissions at 530 nm from free CyPet-SUMO1 and YPet-M1 need to be determined and subtracted from the total emission intensity at 530 nm. To account for the contributions to the total emission at 530 nm, we used a previously established spectrum analysis for determining the EmFRET. The total fluorescent emission at 530 nm given a 414 nm excitation (Em_Total_) was differentiated into three fractions: real FRET emission (EmFRET), CyPet direct emission, and YPet direct emission.

The direct fluorescence contribution of the CyPet at 530 nm was proportional to its peak emission at 475 nm (FL_DD_) when excited at 414 nm with a ratio coefficient of α = 0.368. The direct emission of YPet at 530 nm was proportional to its emission at 530 nm given a 475 nm excitation (FL_AA_) with a ratio coefficient of β = 0.029.

(2) $$Em_{FRET}=Em_{Total}-\alpha *FL_{DD}-\beta *FL_{AA}$$

The Fluorescence signal was compared from 400 nm to 600 nm for each sample. The amount of SUMOylated YPet-M1 was also determined by western blot using an anti-SUMO1 antibody (Santa Cruz Biotechnology, Santa Cruz, CA, USA).

### 2.7. Reconstitution of Wild-Type Influenza B Virus (B/Yamagata/16/1988) and M1 Mutant Influenza B Virus (B/Yamagata/16/1988)

The plasmid-based reverse genetic techniques to generate a recombinant influenza virus have been described in the DNA construct section [36]. To generate the wildtype virus, eight ambisense plasmids encoding the individual segments of the influenza B virus were mixed in 1 mg quantities in 50 mL of serum-free RPMI per transfection. M1 mutant viruses were generated with plasmids containing the mutations instead of the wildtype plasmid. Ten milliliters of lipofectamine 2000 was used per transfection in 250 mL of serum-free RPMI. The plasmid cocktail and lipofectamine were mixed and incubated at room temperature for 15 min before being added to a mixture of MDCK and HEK293 cells. One 10-cm plate of HEK293 and another 10-cm plate of MDCK at 90% confluence were aspirated and washed with 5 mL of 1× PBS, followed by resuspension with 0.25% Trypsin- EDTA. The cells were placed in separate 15 mL conical tubes and centrifuged at 800× *g* for 5 min, and the MDCK cells were resuspended with 1 mL of DMEM with 10% FBS and 1× Pen-Strep Glutamine (Supplemented DMEM). The 1-mL MDCK suspension was then used to resuspend the HEK293 cells and 500 µL of the cell suspension was distributed among two 10-cm tissue culture-treated plates. A volume of 9.5 mL of supplemented DMEM was added to each plate containing cells, the transfection mixture was added to each plate, and the viruses were generated in double. Twenty-four hours after transfection, the transfection medium was replaced with 1× MEM containing 0.3% BSA, 1% Pen-Strep Glutamine, 0.1% FBS, and 1 mg/mL tosylsulfonyl phenylalanyl chloromethyl ketone (TPCK)-trypsin for 72 h at 33°C to produce mature viral particles. The supernatant was then passaged to infect fresh MDCK cells at 90% confluence in a 10-cm plate.

### 2.8. Plaque Assay

Confluent MDCK cells were seeded into each well of 6-well tissue culture-treated plates. After a 24-h culture, the cells were pretreated with different concentrations of STE025/PYR-41 for 24 h. Viruses were serially diluted in a total volume of 300 mL in a medium consisting of 1× PBS, 6.11 mg CaCl_2_ dihydrate, 10.7 mg MgCl_2_ hexahydrate, 0.42% BSA, and 1× Pen-Strep Glutamine (Gibco Ref: 10378-016). Two hundred milliliters of diluted virus samples was added to each plate and incubated at 33 °C for IBV in a humidified 5% CO2 incubator for one hour with rocking every ten minutes. The supernatant was aspirated and 2 mL of plaquing medium (1× EMEM (Gibco Ref: 11430-030), 0.42% BSA, 1× Pen- Strep Glutamine (Gibco Ref: 10378-016), 10 mM HEPES pH 7.4, 0.1 % sodium bicarbonate, 0.1% dextrose, 1 mg/mL TPCK-trypsin and 0.24% Avicel RC-591 NF) and different concentrations of STE025/PYR-41 were added to each well. The plates were incubated in a humidified 5% CO_2_ incubator at 33 °C for 72 h. The cells were then fixed with 1 mL of 4% (*w*/*v*) paraformaldehyde in PBS for one hour and stained with 1 mL of a solution containing 1% crystal violet (*w*/*v*) in 10% methanol (*v*/*v*).

### 2.9. Natural Red Assay

The Neutral Red Cell Cytotoxicity Assay Kit (Bio Vision Catalog # K447-1000) was used in this experiment. A total of 1 × 10^4^ MDCK cells per well were seeded in a 96-well plate one day before performing the Natural red assay. The experiment was divided into four conditions (Virus only, Virus with different concentrations of PYR-41, Virus with different concentrations of STE025, and the control). After a 24-h culture, half of the plate was pretreated with different concentrations of specific drug overnight. The culture media was removed and washed with 1X PBS. Virus samples were diluted in 1X PBS with Pen strep glutamine, CaCl_2_-2H_2_O/MgCl_2_-6H_2_O 100X, and 35% BSA. The appropriate dilution, which made the MOI reach 0.01, was added to the corresponding well and incubated for 72 h at 33 °C and 5% CO_2_. The culture media were removed and washed with a 1X washing buffer. The plates were then stained with a 100X neutral red staining solution for approximately 2h at 37 ℃ in a 5% CO_2_ incubator. The neutral red staining solution was removed by complete aspiration, and the cells were rinsed with a 1X washing buffer to remove the residual dye. The washing buffer was completely removed, and the incorporated neutral red was eluted with a 1X solubilization solution for 30 min. The dye content in each well was quantified using FlexstationII^384^ (Molecular Devices, Sunnyvale, CA, USA) at a wavelength of 540 nm. The dye content in each set of wells was converted to a percentage of the dye range present between the virus control and the untreated control wells using a Microsoft Excel computer-based spreadsheet.

## 3. Results

### 3.1. Identification of the Human SUMOylation Pathway as a Critical Host Pathway for IBV’s Life Cycle

#### 3.1.1. IBV Is Completely Inhibited by the SUMOylation Specific Inhibitor

Many host-viral interactions have been reported before, including those in influenza viruses and coronaviruses [43,44,45]. In previous genome-wide siRNA or CRISPR screenings, SUMOylation enzymes were found to contribute to the IAV life cycle; moreover, many IAV proteins are SUMOylated [26,27,29]. We also recently identified important SUMOylation sites of IAV NS1, whose mutation significantly slows down the IAV growth [33]. More significantly, we recently discovered that IAV could be completely inhibited by a SUMOylation-specific inhibitor, STE025, which inhibited the E1 enzyme of SUMOylation and was discovered through a high-throughput screening campaign, and a critical SUMOylation site of IAV M1 protein was identified throughout the FRET-MS approach (Way et al., submitted). However, although there may be some significant similarities between IAV and IBV, the circulation and clinical outcomes of IBV infections are significantly different. IBV has not been studied as extensively as IAV, and it would be interesting to dissect the host–IBV interactions and to harness this information for new therapeutic developments.

**Figure 1:**
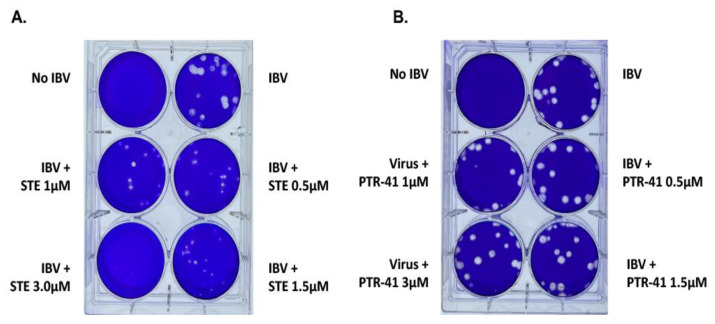
Inhibition of the IBV by the SUMOylation-specific inhibitor, but not the Ubiquitin inhibitor. (**A**) The IBV was inhibited by the specific SUMOylation inhibitor, STE025, in a dose-dependent manner. The MDCK cells were infected with IBV viruses for three days before the plaque staining. (**B**) The IBV virus was not inhibited by the Ubiquitin specific inhibitor, PTR-41.

We first determined whether the IBV was sensitive to the SUMOylation-specific inhibitor, STE025. Due to the lethality of SUMO E1, E2, and multiple E3 gene-knockout mice, it is not feasible to determine the role(s) of SUMOylation in many physiological or pathological processes. We, therefore, developed a FRET-based high-throughput screening to discover small molecule inhibitor(s) for SUMOylation to dissect the roles of SUMOylation. After screening for more than 220,000 compounds and validating hits, we identified a specific SUMOylation inhibitor, STE025, but not for ubiquitination or NEDDylation (Way, G., submitted). We first tested the potential antiviral activity of STE025 for IBV in a plaque assay. Significantly, the STE025 could almost completely inhibit IBV growth at the concentration of 3.5µM, indicating an excellent inhibitory activity of the STE025 for IBV replication (Figure 1A). This result was similar to our previous study of the SUMOylation inhibitor for the IAV, suggesting that the SUMOylation could be a conserved required host pathway for both the IAV and the IBV.

The SUMOylation is a Ubiquitin-like cascade that adds SUMO peptide to the target proteins to regulate their activities and sometimes to compete with the Ubiquitin-mediated protein degradation. To determine whether IBV replication also requires ubiquitination, we used a ubiquitination small molecule inhibitor, PTR-41, which explicitly inhibits the Ubiquitin-Activating Enzyme (E1) [46]. Our results showed no inhibitory activity of PTR-41 for the IBV life cycle (Figure 1B). This result suggests that the IBV may require SUMOylation, but not ubiquitination, for its infection cycle.

**Figure 2:**
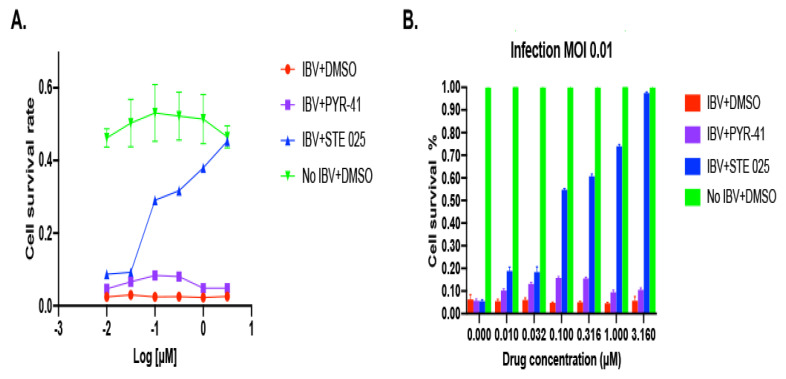
Cell death induced by IBV infection can be rescued by the SUMOylation-specific inhibitor, STE025. (**A**) The MDCK cells were incubated overnight with the SUMOylation-specific inhibitor, STE025, before infection with IBV for 3 days. The cell survival was determined using a Neutral Red assay. The death of MDCK cells infected with IBV was completely rescued with the STE025, but not PTY-41. (**B**) The percentages of cell rescue were calculated according to the control group cells with no virus infection.

We then determined the potency of the SUMOylation inhibitor, STE025, for the IBV inhibition. The IC_50_ of the STE025 inhibition for the SUMOylation is approximately 0.8 µM in a biochemical SUMOylation reaction. A cell-based assay of cell death induced by IBV was set up to determine the potency of the STE025 for IBV inhibition using the neutral red cell cytotoxicity assay. MCDK cells were plated in a 96-well plate the day before a pre-treatment of the STE025 or as a control, PTY-41. The cells were infected with IBV viruses for three days before the cell toxicities were determined. Compared with the cells infected by IBV (red line), the STE025 rescued IBV-infected cells from death in a dose-dependent mode (Figure 2A). Not surprisingly, the ubiquitination inhibitor, PTY-41, did not affect cell death from IBV infection. The EC_50_ of the STE025 for the inhibition of IBV-induced cell death was approximately 0.1 µM, much lower than its IC_50_ in vitro (Figure 2B blue bar). Although the EC_50_ is often different from the IC_50_ values for pharmacological probes or drugs, the significantly lower EC_50_ of the STE025 in the inhibition of IBV-induced cell death than its IC_50_ suggests that the SUMOylation may not need to be completely inhibited in vivo for its inhibitory role in IBV’s life cycle. This hypothesis can be partially verified through the determinations of the SUMOylation enzyme-substrate interaction affinities.

#### 3.1.2. IBV M1 Protein as a Target of SUMOylation

As many IAV proteins have been reported to be SUMOylated and some have effects on virus growth, none of them has been shown to be essential for the viral life cycle. Therefore, we initiated extensive efforts to search for SUMOylation sites and their functional elucidations. In one of these efforts, we combined SUMOylation site prediction algorithms followed with an in vitro SUMOylation assay to determine the SUMOylation sites of NS1 systematically. After SUMOylation assay validation, we found a new SUMOylation site, Lys131. The mutation of NS1 K131A significantly reduces the viral growth rate [33]. Because SUMOylation can be easily removed during cell lysis, we believe an in vitro SUMOylation assay, including SUMOylation E1, E2, and E3, may be more comprehensive. 

**Figure 3:**
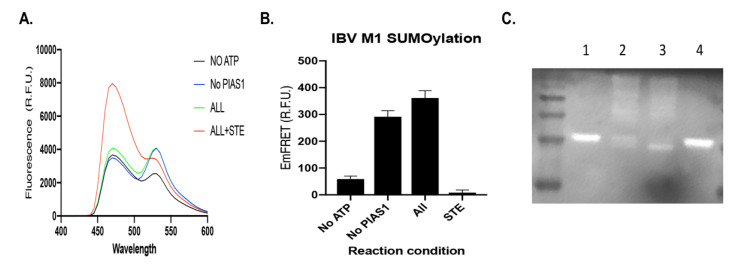
The M1 protein of IBV was SUMOylated and the SUMOylation of the M1 protein was inhibited with the SUMOylation inhibitor STE025 in both vitro FRET assay and the biochemical assay. (**A**) The FRET spectrum of the in vitro SUMOylation reaction of IBV M1 protein using the FRET assay. Four reactions were conducted, CyPet-SUMO1, E1, E2, E3, YPet-M1, and ATP (ALL and green); CyPet-SUMO1, E1, E2, YPet-M1, and ATP (no PIAS1 and Blue); E1, E2, E3, YPet-M1 and no ATP (NO ATP and black); CyPet-SUMO1, E1, E2, E3, YPet-M1, ATP, and STE (ALL plus STE and red). (**B**) Quantitative FRET signal (EmFRET) of IBV M1 SUMOylation from (**A**). (**C**) In vitro biochemical assay of the SUMOylation of IBV M1 protein followed with a Western blot using anti-SUMO1 antibody. The SUMOylation reactions were conducted in solution in various conditions with or without the SUMOylation inhibitor, STE. Lane 1, CyPet-SUMO1, E1, E2, E3, YPet-M1, -ATP; Lane 2, CyPet-SUMO1, E1, E2, YPet-M1, +ATP; Lane 3, CyPet-SUMO1 1, E1, E2, E3, YPet-M1, +ATP; Lane 4, CyPet-SUMO1, E1, E2, YPet-M1, +ATP+STE025.

The SUMOylation of IBV proteins has not been explored yet, and therefore we set up an in vitro SUMOylation assay for IBV proteins. The M1 protein plays a critical role in viral RNA trafficking and assembly, and the M1 protein of IAV is SUMOylated [27,31] (Way, G. et al., submitted). The M1 protein of IBV was first fused with the FRET pair acceptor YPet as YPet-M1 to act as substrate in the SUMOylation assay to receive the FRET donor, CyPet-SUMO1, after cascade enzymatic reactions. After the CyPet-SUMO1’s conjugation to the YPet-M1, due to the proximity of the FRET donor and acceptor, the FRET signal was very significant (Figure 3A ALL green). This SUMOylation reaction was also inhibited by the inhibitor, STE025, as monitored with the FRET signal (Figure 3A ALL+STE025 and red). In this SUMOylation assay, we also included SUMOylation E3 ligase PIAS1 in the reaction. The SUMOylation E3 ligase has been suggested to play an important role in in vivo SUMOylation when protein concentrations are not very high, but it is not needed for the in vitro SUMOylation reaction. We did not observe too much FRET signal increase after the addition of PIAS1 to the reaction, which agreed with the general common knowledge (Figure 3A No PIAS1 blue). However, after a careful quantification of the FRET signal through our qFRET method, the SUMOylation reaction produced higher signals than the one without PIAS1 (Figure 3B) [47]. The SUMOylation reaction monitored with the FRET signal was a convenient method for following the reaction and obtaining results. To validate the FRET-monitored SUMOylation reaction, we also performed a classical SUMOylation reaction followed by a Western blot assay. The SUMOylation reaction included components of CyPet-SUMO1, E1, E2, E3, YPet-M1, +/-ATP, or +/-STE025. No SUMOylation reaction occurred if all other components were presented but no ATP (Figure 3C, lane 1). The SUMOylation reactions occurred with or without E3 PIAS1, and the SUMOylated substrate YPet-M1 was shown in higher molecular weight ladders (Figure 3C Lane 2 and 3, respectively). The SUMOylation reaction was also inhibited by the SUMOylation inhibitor STE025 (Figure 3C Lane 4). These results confirmed the conclusions from the FRET-monitored SUMOylation reactions in Figure 3A, suggesting the SUMOylation reaction monitored with the FRET signal was a good approach. These results also indicate that IBV M1 is SUMOylated, and this SUMOylation may have some roles in the M1 activity and later viral life cycle.

#### 3.1.3. Recognition of IBV M1 Protein by the SUMOylation E2 and E3 with High Affinity

The SUMOylation of proteins is mediated through an enzymatic cascade involving an E1 activating enzyme, namely Aos1/Uba2, an E2 conjugating enzyme, namely Ubc9, and an E3 ligating enzyme, such as the proteins from the PIAS family [48,49]. It is generally assumed that the in vitro SUMOylation does not need E3, and E3 plays more critical roles in terms of efficiency and specificity in vivo [50,51,52]. It will be interesting to determine the affinities of SUMOylation E2 and E3 to the IBV M1 protein, a heterologous protein for humans.

**Figure 4:**
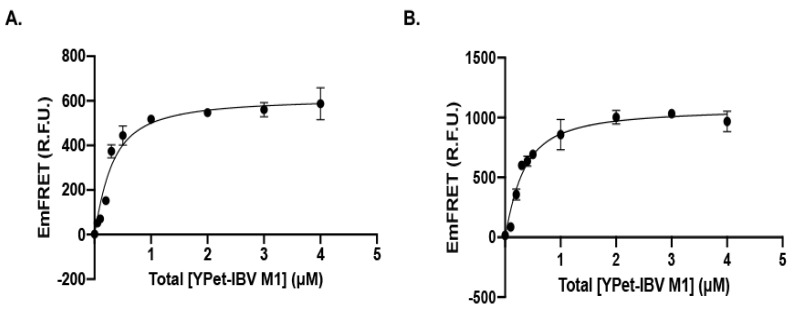
The SUMOylation E2 conjugation enzyme, Ubc9, and E3 ligase, PIAS1, interact with M1 protein of IBV at a very high affinity determined with a qFRET assay (**A**). The interaction affinity K_D_ value of 0.2 µM between Ubc9 and M1 was determined using the qFRET assay in solution (**B**). The interaction affinity K_D_ value of 0.22µM between PIAS1 and M1 was determined using the qFRET assay.

We applied our FRET-based K_D_ determination technology to the determinations of affinities between Ubc9 or PIAS1 to the IBV M1 protein [42,47,53]. The Ubc9 or PIAS1 genes were first fused with the FRET donor, CyPet, and IBV M1 was fused with the FRET acceptor, YPet. The fluorescent proteins were expressed in *E. coli* cells Bl_21_ (DE3) and purified through the Ni-His affinity column. Then, in a fixed concentration of the FRET donor, the CyPet-Ubc9 or the CyPet-PIAS1, different concentrations of the FRET acceptor, the YPet-M1, were titrated. We have developed an algorithm to extract the absolute FRET signal, which corresponded to the interactions, from the total fluorescence signal [47]. The titrated absolute fluorescence signal (Em_FRET_) for Ubc9 and PIAS1 with M1 showed good sigmoidal curves (Figure 4A,B). The K_D_ values for Ubc9-M1 or PIAS1-M1 interactions were 0.20 µM and 0.22 µM, respectively, indicating a very high affinity of both SUMOylation enzymes E2 and E3 for the IBV M1. These K_D_ values are equivalent or lower than those of cellular substrates, such as PCNA or Ran Gap1, suggesting that the IBV M1 protein is an excellent substrate of SUMOylation.

#### 3.1.4. Identification of an Essential SUMOylation Site of the IBV M1 Protein

Although many viral proteins, including the IAV, have been determined to be SUMOylated, only a small fraction of these SUMOylation sites have been shown to play roles in the viral life cycle. None of the studied SUMOylation sites are essential for the viral life cycle of IAV [26,28,33,54].

**Figure 5:**
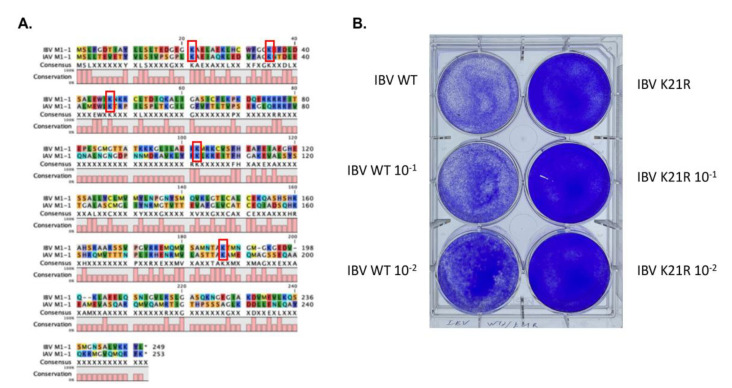
Identification of essential SUMOylation site Lys21 of M1 for the IBV life cycle. (**A**) The alignment of IBV and IAV M1 proteins. Five Lys residues were conserved between the IBV and IAV M1 proteins, Lys21, Lys35, Ly46, Lys102, and Lys187. (**B**) The M1 Lys21 mutation of IBV abolishes the viral life cycle. The IBV virus with a M1 K21R mutation could not produce a viral particle, while the wild-type IBV virus could be generated and killed the cells.

In another, more comprehensive search for SUMOylation site(s) of the IAV M1 protein through a combination approach of FRET-SUMOylation-MS, we found that several sites of IAV M1 were SUMOylated, including Lys 21, Ly35, Lys 187, Lys 230, Lys 242, and Lys 252 (Way, G. et al., submitted). We mutated each of the above Lys in the IAV M1 gene, and we found that the Lys21Arg mutation completely abolished viral replication. IBV is similar to IAV in many aspects, from the genome to transmission and pathology. We speculated that viral interactions with the host might be similar to some degree. We first aligned the IBV M1 and IAV M1 proteins and found a very low similarity (Figure 5A). However, five Lys residues, Lys21, Lys35, Ly46, Lys102, and Lys187, were conserved. Because the Lys35Arg and Lys187Arg did not show any significant impact on the viral life cycle, we then tested the M1 Lys21 mutation in IBV replication. The M1 Lys21Arg mutation completely abolished IBV rescue in a plasmid transfection rescue/plaque assay (Figure 5B). This result strongly suggests that SUMOylation on Lys21 is critical for the IBV M1 activities, and that SUMOylation is critical for the IBV life cycle.

## 4. Discussion

Viruses infect hosts, such as humans, by taking advantage of host factors for their life cycles. The roles of these factors are different depending on whether they can be replaced by other host factors or not. Here, for the first time, we determine that the human SUMOylation pathway is essential for the IBV life cycle. IBV virus replication can be inhibited by the novel SUMOylation inhibitor, STE025, in a plaque assay. The STE025 has a very high inhibition potency, at about 0.1 µM in the rescuing IBV-induced cell death assay. This potency is higher than its IC_50_ in in vitro SUMOylation assay, suggesting a good potential in in vivo applications. The mechanism underlining the viral inhibition by the SUMOylation inhibitor STE025 is at least partly due to the requirement of SUMOylation of the IBV M1 protein. The IBV M1 protein is recognized by the SUMOylation E2 conjugation enzyme and E3 ligase at very high affinities, both around 0.2 µM in our qFRET assay, suggesting the SUMOylation of M1 under in vivo physiological conditions. The detailed functional requirement of SUMOylation for the M1 protein needs to be further explored, but this protein is known to play essential roles in viral RNA trafficking and in viral assembly. In addition, the clinical data showed that children were more susceptible and had a higher rate of hospitalization when infected with IBV than IAV, reflecting different pathological processes and immune responses caused by IBV infection [7]. In a cellular assay, IBV was revealed to be less sensitive to the oseltamivir than IAV [55]. Although the detail mechanisms underlying the differences of IBV and IAV need more studies, this suggests that anti-flu drug discoveries should also consider activities in IBV.

The SUMO E3 ligase family of PIAS was first discovered through a yeast two-hybrid screening for its interactions with STAT proteins. The overexpression of PIAS genes inhibits cytokine signaling, which is critical in innate immune responses against viral infections [56,57,58]. Thus, the inhibition of PIAS results in increased antiviral responses. In addition to therapies based on increasing the host’s antiviral immune responses, the dissection of the direct virus–host interactions provides opportunities for the development of anti-viral therapeutics. First, many viruses, such as Ebola, CMV, and EMCV, utilize SUMOylation to inhibit anti-viral intrinsic and innate immunity, such as reducing interferon productions and inhibiting STAT1/3, PML, IRFs, and NFkB [26,54,56,57,59,60,61,62,63,64]. For example, the Ebola viral VP35 interacts with interferon regulatory factors (IRF)-3/7 and PIAS1, resulting in their SUMOylation and their transcriptional repression, thereby suppressing the production of type-I interferons [61]. Other viruses, such as SARS-CoV-2, SARS, and influenza viruses, also suppress IFN I production and signaling [65,66,67,68]. Significant clinical improvements have been observed in a clinical trial for the treatment of moderate to severe COVID-19 patients using an engineered IFNα, rSIFN-co, strongly suggesting that IFNα antagonized by viruses is critical for evading the host’s immune response against viral infection [69]. Importantly, many viruses manipulate the SUMOylation process to ensure their propagation and survival. Herpes simplex virus-1 (HSV-1) infection results in a three-fold decrease in the modification of over 100 cellular proteins, including the anti-viral promyelocytic leukemia (PML) nuclear bodies PML [63]. IAV infection results in the increased SUMOylation of nearly 200 cellular proteins and the de-SUMOylation of over 500 cellular proteins [27,70]. Moreover, viruses directly manipulate the SUMOylation machinery for their own benefit. The human adenovirus protein, E1A, blocks the interaction of UBC9 and substrates, resulting in the inhibition of the SUMOylation of the target proteins [65]. There are also examples of viruses that take advantage of host post-translational modifications, such as SUMOylation, to induce conformational changes of target proteins, to enhance their functionalities. For example, the immediate-early protein Rta of the Epstein–Barr virus (EBV), which activates the transcription of EBV lytic genes and the lytic cycle, is enhanced after SUMOylation [66]. Finally, almost all transcriptional factors involved in viral sensing, innate and adaptive immunities, such as IRF, STAT, and NFkB, are negatively regulated by SUMOylation [56,57]. Therefore, all of this supports the notion that SUMOylation inhibition can be an attractive strategy of antiviruses.

Targeting host factors for the treatment of viral infections can bring potential additional important benefits of reduced drug resistance. There may be several benefits to targeting host factors. First, the viral genomes, particularly ssRNA viruses, such as influenza viruses, have a very high mutation rate (10^−4^–10^−6^ substitutions/bp/cell infection) mainly due to the lack of proofreading activities of their RNA-dependent RNA Polymerases (RdRP) and RNA-dependent DNA Polymerases (RdDP) or Reverse Transcriptases (RTase), [71,72,73,74]. In contrast, the mutation rate of DNA polymerases is much lower (10^−9^–10^−10^ substitutions/bp/cell division), mostly due to both the proofreading activity of DNA polymerase and the mismatch repair pathways of host cells [72,73,75,76]. Therefore, the spontaneous mutation rate of viral genes is much higher than the host genes, leading to a high probability of drug resistance development when viral genes are targeted for therapy [77].

## 5. Conclusions

A new approach to targeting host factors for anti-pathogens has emerged as a new strategy for anti-viral therapeutics [38,39,43,44,74,78]. For example, antibodies blocking the human receptor ACE2 of SARS-CoV-2 have shown efficacy in protecting SARS-CoV-2 patients [79]. We demonstrate here the possibility of inhibiting the human SUMOylation pathway as a novel approach for anti-IBV infection therapy, which agrees with others and our hypothesis [28,33,39,80]. The discovery and characterization of cellular factors or pathways critical for the pathogen life cycle in a host or that regulate pathogenesis holds great promise for revealing new strategies for anti-infectives.

## 6. Patents

The SUMOylation inhibitor and its application in anti-viruses and anti-cancers was patented by the University of California.

## Data Availability

All the data can be available upon request to J.L. at Jiayu.liao@ucr.edu.

## References

[1] Centers for Disease Control and Prevention (2019). Estimated Flu-Related Illnesses, Medical Visits, Hospitalizations, and Deaths in the United States—2017–2018 Flu Season. https://www.cdc.gov/flu/about/burden/2018-2019.html.

[2] Putri W., Muscatello D.J., Stockwell M.S., Newall A.T. (2018). Economic burden of seasonal influenza in the United States. Vaccine.

[3] Rota P.A., Wallis T.R., Harmon M.W., Rota J.S., Kendal A.P., Nerome K. (1990). Cocirculation of two distinct evolutionary lineages of influenza type B virus since 1983. Virology.

[4] Yan S., Weycker D., Sokolowski S. (2017). US healthcare costs attributable to type A and type B influenza. Hum. Vaccines Immunother..

[5] Mosnier A., Caini S., Daviaud I., Nauleau E., Bui T.T., Debost E., Bedouret B., Agius G., van der Werf S., Lina B. (2015). Clinical Characteristics Are Similar across Type A and B Influenza Virus Infections. PLoS ONE.

[6] Bhat Y.R. (2020). Influenza B infections in children: A review. World J. Clin. Pediatr..

[7] Tran D., Vaudry W., Moore D., Bettinger J.A., Halperin S.A., Scheifele D.W., Jadvji T., Lee L., Mersereau T., members of the Canadian Immunization Monitoring Program, A (2016). Hospitalization for Influenza A Versus B. Pediatrics.

[8] Cohen C., Moyes J., Tempia S., Groom M., Walaza S., Pretorius M., Dawood H., Chhagan M., Haffejee S., Variava E. (2013). Severe influenza-associated respiratory infection in high HIV prevalence setting, South Africa, 2009–2011. Emerg. Infect. Dis..

[9] Centers for Disease Control and Prevention (2011). Influenza-Associated Pediatric Deaths—United States, September 2010–August 2011. MMWR Morb. Mortal. Wkly. Rep..

[10] Centers for Disease Control and Prevention Quadrivalent Influenza Vaccine. https://www.cdc.gov/flu/prevent/quadrivalent.htm.

[11] Centers for Disease Control and Prevention US Flu VE Data for 2019–2020. https://www.cdc.gov/flu/about/burden/2019-2020.html.

[12] Monto A.S., McKimm-Breschkin J.L., Macken C., Hampson A.W., Hay A., Klimov A., Tashiro M., Webster R.G., Aymard M., Hayden F.G. (2006). Detection of influenza viruses resistant to neuraminidase inhibitors in global surveillance during the first 3 years of their use. Antimicrob. Agents Chemother..

[13] Sheu T.G., Deyde V.M., Okomo-Adhiambo M., Garten R.J., Xu X., Bright R.A., Butler E.N., Wallis T.R., Klimov A.I., Gubareva L.V. (2008). Surveillance for neuraminidase inhibitor resistance among human influenza A and B viruses circulating worldwide from 2004 to 2008. Antimicrob. Agents Chemother..

[14] Dharan N.J., Gubareva L.V., Meyer J.J., Okomo-Adhiambo M., McClinton R.C., Marshall S.A., St George K., Epperson S., Brammer L., Klimov A.I. (2009). Infections with oseltamivir-resistant influenza A(H1N1) virus in the United States. JAMA.

[15] Li X., Liao H., Liu Y., Liu L., Wang F., Song H., Cheng J., Liu X., Xu D. (2017). Drug-Resistant and Genetic Evolutionary Analysis of Influenza Virus from Patients During the 2013 and 2014 Influenza Season in Beijing. Microb. Drug Resist..

[16] Matsuzaki Y., Mizuta K., Aoki Y., Suto A., Abiko C., Sanjoh K., Sugawara K., Takashita E., Itagaki T., Katsushima Y. (2010). A two-year survey of the oseltamivir-resistant influenza A(H1N1) virus in Yamagata, Japan and the clinical effectiveness of oseltamivir and zanamivir. Virol. J..

[17] Hatakeyama S., Sugaya N., Ito M., Yamazaki M., Ichikawa M., Kimura K., Kiso M., Shimizu H., Kawakami C., Koike K. (2007). Emergence of influenza B viruses with reduced sensitivity to neuraminidase inhibitors. JAMA.

[18] Abed Y., Fage C., Lague P., Carbonneau J., Papenburg J., Vinh D.C., Boivin G. (2019). Reduced Susceptibility to Neuraminidase Inhibitors in Influenza B Isolate, Canada. Emerg. Infect. Dis..

[19] Kawai N., Ikematsu H., Iwaki N., Kawashima T., Maeda T., Mitsuoka S., Kondou K., Satoh I., Miyachi K., Yamaga S. (2007). Longer virus shedding in influenza B than in influenza A among outpatients treated with oseltamivir. J. Infect..

[20] Jefferson T., Jones M.A., Doshi P., Del Mar C.B., Hama R., Thompson M.J., Spencer E.A., Onakpoya I., Mahtani K.R., Nunan D. (2014). Neuraminidase inhibitors for preventing and treating influenza in adults and children. Cochrane Database Syst. Rev..

[21] Ison M.G., Portsmouth S., Yoshida Y., Shishido T., Mitchener M., Tsuchiya K., Uehara T., Hayden F.G. (2020). Early treatment with baloxavir marboxil in high-risk adolescent and adult outpatients with uncomplicated influenza (CAPSTONE-2): A randomised, placebo-controlled, phase 3 trial. Lancet Infect. Dis..

[22] Konig R., Stertz S., Zhou Y., Inoue A., Hoffmann H.H., Bhattacharyya S., Alamares J.G., Tscherne D.M., Ortigoza M.B., Liang Y. (2010). Human host factors required for influenza virus replication. Nature.

[23] Karlas A., Machuy N., Shin Y., Pleissner K.P., Artarini A., Heuer D., Becker D., Khalil H., Ogilvie L.A., Hess S. (2010). Genome-wide RNAi screen identifies human host factors crucial for influenza virus replication. Nature.

[24] Han J., Perez J.T., Chen C., Li Y., Benitez A., Kandasamy M., Lee Y., Andrade J., tenOever B., Manicassamy B. (2018). Genome-wide CRISPR/Cas9 Screen Identifies Host Factors Essential for Influenza Virus Replication. Cell Rep..

[25] Liu B., Mink S., Wong K.A., Stein N., Getman C., Dempsey P.W., Wu H., Shuai K. (2004). PIAS1 selectively inhibits interferon-inducible genes and is important in innate immunity. Nat. Immunol..

[26] Everett R.D., Boutell C., Hale B.G. (2013). Interplay between viruses and host sumoylation pathways. Nat. Rev. Microbiol..

[27] Pal S., Santos A., Rosas J.M., Ortiz-Guzman J., Rosas-Acosta G. (2011). Influenza A virus interacts extensively with the cellular SUMOylation system during infection. Virus Res..

[28] Wimmer P., Schreiner S., Dobner T. (2012). Human pathogens and the host cell SUMOylation system. J. Virol..

[29] Boggio R., Chiocca S. (2006). Viruses and sumoylation: Recent highlights. Curr. Opin. Microbiol..

[30] Xu K., Klenk C., Liu B., Keiner B., Cheng J., Zheng B.J., Li L., Han Q., Wang C., Li T. (2011). Modification of nonstructural protein 1 of influenza A virus by SUMO1. J. Virol..

[31] Wu C.Y., Jeng K.S., Lai M.M. (2011). The SUMOylation of matrix protein M1 modulates the assembly and morphogenesis of influenza A virus. J. Virol..

[32] Han Q., Chang C., Li L., Klenk C., Cheng J., Chen Y., Xia N., Shu Y., Chen Z., Gabriel G. (2014). Sumoylation of influenza A virus nucleoprotein is essential for intracellular trafficking and virus growth. J. Virol..

[33] Way G., Xiong Z., Wang G., Dai H., Zheng S., Garcia-Sastre A., Liao J. (2020). A novel SUMOylation site in the influenza a virus NS1 protein identified with a highly sensitive FRET assay. J. Biotechnol..

[34] Furuse Y., Suzuki A., Kamigaki T., Oshitani H. (2009). Evolution of the M gene of the influenza A virus in different host species: Large-scale sequence analysis. Virol. J..

[35] Ito T., Gorman O.T., Kawaoka Y., Bean W.J., Webster R.G. (1991). Evolutionary analysis of the influenza A virus M gene with comparison of the M1 and M2 proteins. J. Virol..

[36] Koelle K., Cobey S., Grenfell B., Pascual M. (2006). Epochal evolution shapes the phylodynamics of interpandemic influenza A (H3N2) in humans. Science.

[37] Choo J.A., Liu J., Toh X., Grotenbreg G.M., Ren E.C. (2014). The immunodominant influenza A virus M158-66 cytotoxic T lymphocyte epitope exhibits degenerate class I major histocompatibility complex restriction in humans. J. Virol..

[38] Van de Sandt C.E., Kreijtz J.H., Geelhoed-Mieras M.M., Nieuwkoop N.J., Spronken M.I., van de Vijver D.A., Fouchier R.A., Osterhaus A.D., Rimmelzwaan G.F. (2016). Differential Recognition of Influenza A Viruses by M158-66 Epitope-Specific CD8^+^ T Cells Is Determined by Extraepitopic Amino Acid Residues. J. Virol..

[39] Song Y., Liao J. (2012). Systematic determinations of SUMOylation activation intermediates and dynamics by a sensitive and quantitative FRET assay. Mol. Biosyst..

[40] Malik-Chaudhry H.K., Saavedra A., Liao J. (2014). A linker strategy for trans-FRET assay to determine activation intermediate of NEDDylation cascade. Biotechnol. Bioeng..

[41] Fodor E., Devenish L., Engelhardt O.G., Palese P., Brownlee G.G., Garcia-Sastre A. (1999). Rescue of influenza A virus from recombinant DNA. J. Virol..

[42] Song Y., Madahar V., Liao J. (2011). Development of FRET Assay into Quantitative and High-throughput Screening Technology Platforms for Protein-Protein Interactions. Ann. Biomed. Eng..

[43] Prussia A., Thepchatri P., Snyder J.P., Plemper R.K. (2011). Systematic approaches towards the development of host-directed antiviral therapeutics. Int. J. Mol. Sci..

[44] Kellam P. (2006). Attacking pathogens through their hosts. Genome Biol..

[45] De Chassey B., Meyniel-Schicklin L., Vonderscher J., Andre P., Lotteau V. (2014). Virus-host interactomics: New insights and opportunities for antiviral drug discovery. Genome Med..

[46] Yang Y., Kitagaki J., Dai R.M., Tsai Y.C., Lorick K.L., Ludwig R.L., Pierre S.A., Jensen J.P., Davydov I.V., Oberoi P. (2007). Inhibitors of Ubiquitin-Activating Enzyme (E1), a New Class of Potential Cancer Therapeutics. Cancer Res..

[47] Liao J., Madahar V., Dang R., Jiang L. (2021). Quantitative FRET (qFRET) Technology for the Determination of Protein-Protein Interaction Affinity in Solution. Molecules.

[48] Kerscher O., Felberbaum R., Hochstrasser M. (2006). Modification of proteins by ubiquitin and ubiquitin-like proteins. Annu. Rev. Cell Dev. Biol..

[49] Sarge K.D., Park-Sarge O.K. (2011). SUMO and its role in human diseases. Int. Rev. Cell Mol. Biol..

[50] Reverter D., Lima C.D. (2005). Insights into E3 ligase activity revealed by a SUMO-RanGAP1-Ubc9-Nup358 complex. Nature.

[51] Yunus A.A., Lima C.D. (2009). Structure of the Siz/PIAS SUMO E3 ligase Siz1 and determinants required for SUMO modification of PCNA. Mol. Cell.

[52] Pichler A., Fatouros C., Lee H., Eisenhardt N. (2017). SUMO conjugation—A mechanistic view. Biomol. Concepts.

[53] Song Y., Rodgers V.G., Schultz J.S., Liao J. (2012). Protein interaction affinity determination by quantitative FRET technology. Biotechnol. Bioeng..

[54] Lowrey A.J., Cramblet W., Bentz G. (2017). Viral manipulation of the cellular sumoylation machinery. Cell Commun. Signal..

[55] Ferraris O., Kessler N., Lina B. (2005). Sensitivity of influenza viruses to zanamivir and oseltamivir: A study performed on viruses circulating in France prior to the introduction of neuraminidase inhibitors in clinical practice. Antivir. Res..

[56] Chung C.D., Liao J., Liu B., Rao X., Jay P., Berta P., Shuai K. (1997). Specific Inhibition of Stat3 Signal Transduction by PIAS3. Science.

[57] Liu B., Liao J., Rao X., Kushner S.A., Chung C.D., Chang D.D., Shuai K. (1998). Inhibition of Stat1-mediated gene activation by PIAS1. Proc. Natl. Acad. Sci. USA.

[58] Liao J., Fu Y., Shuai K. (2000). Distinct roles of the NH2- and COOH-terminal domains of the protein inhibitor of activated signal transducer and activator of transcription (STAT) 1 (PIAS1) in cytokine-induced PIAS1-Stat1 interaction. Proc. Natl. Acad. Sci. USA.

[59] Hochstrasser M. (2009). Origin and function of ubiquitin-like proteins. Nature.

[60] Baz-Martinez M., El Motiam A., Ruibal P., Condezo G.N., de la Cruz-Herrera C.F., Lang V., Collado M., San Martin C., Rodriguez M.S., Munoz-Fontela C. (2016). Regulation of Ebola virus VP40 matrix protein by SUMO. Sci. Rep..

[61] Chang T.H., Kubota T., Matsuoka M., Jones S., Bradfute S.B., Bray M., Ozato K. (2009). Ebola Zaire virus blocks type I interferon production by exploiting the host SUMO modification machinery. PLoS Pathog..

[62] Bentz G.L., Shackelford J., Pagano J.S. (2012). Epstein-Barr virus latent membrane protein 1 regulates the function of interferon regulatory factor 7 by inducing its sumoylation. J. Virol..

[63] Sloan E., Tatham M.H., Groslambert M., Glass M., Orr A., Hay R.T., Everett R.D. (2015). Analysis of the SUMO2 Proteome during HSV-1 Infection. PLoS Pathog..

[64] Kubota T., Matsuoka M., Chang T.H., Tailor P., Sasaki T., Tashiro M., Kato A., Ozato K. (2008). Virus infection triggers SUMOylation of IRF3 and IRF7, leading to the negative regulation of type I interferon gene expression. J. Biol. Chem..

[65] Channappanavar R., Fehr A.R., Vijay R., Mack M., Zhao J., Meyerholz D.K., Perlman S. (2016). Dysregulated Type I Interferon and Inflammatory Monocyte-Macrophage Responses Cause Lethal Pneumonia in SARS-CoV-Infected Mice. Cell Host Microbe.

[66] Garcia-Sastre A., Egorov A., Matassov D., Brandt S., Levy D.E., Durbin J.E., Palese P., Muster T. (1998). Influenza A virus lacking the NS1 gene replicates in interferon-deficient systems. Virology.

[67] Riva L., Yuan S., Yin X., Martin-Sancho L., Matsunaga N., Pache L., Burgstaller-Muehlbacher S., De Jesus P.D., Teriete P., Hull M.V. (2020). Discovery of SARS-CoV-2 antiviral drugs through large-scale compound repurposing. Nature.

[68] Blanco-Melo D., Nilsson-Payant B.E., Liu W.C., Uhl S., Hoagland D., Moller R., Jordan T.X., Oishi K., Panis M., Sachs D. (2020). Imbalanced Host Response to SARS-CoV-2 Drives Development of COVID-19. Cell.

[69] Li C., Luo F., Liu C., Xiong N., Xu Z., Zhang W., Yang M., Wang Y., Liu D., Yu C. (2021). Effect of a genetically engineered interferon-alpha versus traditional interferon-alpha in the treatment of moderate-to-severe COVID-19: A randomised clinical trial. Ann. Med..

[70] Domingues P., Golebiowski F., Tatham M.H., Lopes A.M., Taggart A., Hay R.T., Hale B.G. (2015). Global Reprogramming of Host SUMOylation during Influenza Virus Infection. Cell Rep..

[71] Sanjuan R., Nebot M.R., Chirico N., Mansky L.M., Belshaw R. (2010). Viral mutation rates. J. Virol..

[72] Duffy S., Shackelton L.A., Holmes E.C. (2008). Rates of evolutionary change in viruses: Patterns and determinants. Nat. Rev. Genet..

[73] Drake J.W. (1993). Rates of spontaneous mutation among RNA viruses. Proc. Natl. Acad. Sci. USA.

[74] Denison M.R., Graham R.L., Donaldson E.F., Eckerle L.D., Baric R.S. (2011). Coronaviruses: An RNA proofreading machine regulates replication fidelity and diversity. RNA Biol..

[75] Drake J.W., Charlesworth B., Charlesworth D., Crow J.F. (1998). Rates of spontaneous mutation. Genetics.

[76] Korona D.A., Lecompte K.G., Pursell Z.F. (2011). The high fidelity and unique error signature of human DNA polymerase epsilon. Nucleic Acids Res..

[77] Liao J., Way G., Madahar V. (2020). Target Virus or Target Ourselves for COVID-19 Drugs Discovery?-Lessons learned from anti-influenzas virus therapies. Med. Drug Discov..

[78] Van de Wakker S.I., Fischer M.J.E., Oosting R.S. (2017). New drug-strategies to tackle viral-host interactions for the treatment of influenza virus infections. Eur. J. Pharmacol..

[79] Taylor P.C., Adams A.C., Hufford M.M., de la Torre I., Winthrop K., Gottlieb R.L. (2021). Neutralizing monoclonal antibodies for treatment of COVID-19. Nat. Rev. Immunol..

[80] Liao J. (1999). Protein Inhibitors of Activated Stat. Ph.D. Thesis.

